# Aptamer-iRNAs as Therapeutics for Cancer Treatment

**DOI:** 10.3390/ph11040108

**Published:** 2018-10-18

**Authors:** Mario M. Soldevilla, Daniel Meraviglia-Crivelli de Caso, Ashwathi P. Menon, Fernando Pastor

**Affiliations:** 1 Molecular Therapy Program, Aptamer Core, Center for the Applied Medical Research (CIMA), University of Navarra (UNAV), 31008 Pamplona, Spain; mmsoldevilla@unav.es (M.M.S.); dmeraviglia@alumni.unav.es (D.M.-C.d.C.); apuravankar@alumni.unav.es (A.P.M.); 2 Navarre Health Research Institute (IdiSNA), 31008 Pamplona, Spain

**Keywords:** aptamer, cancer, iRNA, siRNA, shRNA, microRNA, antagomirs, antisense oligonucleotides, DNA, RNA, therapeutics

## Abstract

Aptamers are single-stranded oligonucleotides (ssDNA or ssRNA) that bind and recognize their targets with high affinity and specificity due to their complex tertiary structure. Aptamers are selected by a method called SELEX (Systematic Evolution of Ligands by EXponential enrichment). This method has allowed the selection of aptamers to different types of molecules. Since then, many aptamers have been described for the potential treatment of several diseases including cancer. It has been described over the last few years that aptamers represent a very useful tool as therapeutics, especially for cancer therapy. Aptamers, thanks to their intrinsic oligonucleotide nature, present inherent advantages over other molecules, such as cell-based products. Owing to their higher tissue penetrability, safer profile, and targeting capacity, aptamers are likely to become a novel platform for the delivery of many different types of therapeutic cargos. Here we focus the review on interfering RNAs (iRNAs) as aptamer-based targeting delivered agents. We have gathered the most reliable information on aptamers as targeting and carrier agents for the specific delivery of siRNAs, shRNA, microRNAs, and antisense oligonucleotides (ASOs) published in the last few years in the context of cancer therapy.

## 1. Introduction

RNA or DNA oligonucleotides are composed of nitrogen bases linked by phosphodiester bonds. Nucleic acids can display encoding, enzymatic, and structural functions, due to which ribonucleotides have been established as the key molecules in the origin of life [1,2]. As a matter of fact, there is a wide variety of functional RNAs in the cell: mRNA, which encodes gene information to be further processed into proteins; viral RNA, which corresponds to the encoding genome of a large family of viruses termed “RNA viruses”; long non-coding RNA (lncRNA), which do not encode for any protein but can display scaffolding structural functions; interference RNA (iRNA), which regulates mRNA translation and comprises small interfering RNAs (siRNAs), short hairpin RNAs (shRNAs), microRNAs, antagomiRs (also called antimiRs), and antisense oligonucleotides (ASOs); structural RNAs, such as ribosomal RNA (rRNA), transference RNAs (tRNAs); and catalytic RNAs with enzymatic activity (ribozymes) [3]. These different types of RNA exert their respective functions by two different modes of action: sequence specific recognition and structural recognition. Structural recognition of nucleic acids is based on pocket interactions implying van der Waals forces, ionic, hydrogen, and other non-covalent bonds. With the broad complexities of structures and function of nucleic acids that are displayed in nature, it is easy to envision that they can be utilized as powerful therapeutic drugs for different purposes [3].

Sequence specific recognition is mediated by a base-pair fashion, coupling complementary sequences to exert its activating or inhibitory function. iRNAs work by sequence specific recognition, base-pairing completely or incompletely (depending on the iRNA subtype) to its target sequence to modulate mRNA translation. RNAi technology is based in posttranscriptional gene silencing (PTGS). PTGS reduces target level transcripts by sequence specific recognition leading to loss of function [4]. The first evidence of iRNA activity in the cells occurred serendipitously in 1990 by Napoli et al. when overexpression of a specific gene in plants (petunia) did not result in the predicted phenotype [5]. This fact led to the discovery of an intrinsic mechanism of defense against potential foreign RNA, providing the first evidence of iRNA existence. This early discovery triggered the development of iRNA therapeutics, which is currently based on siRNAs, shRNAs, microRNAs, ASOs, etc.

iRNAs have been used to modulate mRNA translation in eukaryotic cells [6]. Over time, iRNA technology has been used to silence disease-causing genes when other medicines failed [6,7,8]. They currently provide a powerful tool in cancer therapy by regulating tumor-promoting genes [3,8,9]. Nevertheless, despite their relative success, iRNA-based therapies have encountered several problems in reaching their full potential and keep on going forward as anticancer therapeutics. Biodistribution, stability, off-target effects due to their lack of specificity, and low efficacy at trespassing cell membranes are the major issues limiting iRNA-based therapies in cancer treatment. Due to their proven success as delivery and targeting agents, aptamers present a promising tool to deliver iRNAs to the desired target tissue, increasing their efficacy as anticancer agents. Here we focus this review on aptamer-iRNA chimeras for cancer treatment.

## 2. Aptamers

Aptamers are synthetic single-stranded oligonucleotides (DNA or RNA) of short length (20–100 nucleotides) whose three-dimensional disposition confers them high avidity for their targets. In the early 1990s, the word “aptamer” was coined for the first time by Andrew Ellington and Jack Szostak. They combined the Latin word “aptus” that means “to fit” and the Greek word “meros” that means “particle” to name those selected RNA species that specifically recognized small organic dyes [10]. At the same time Larry Gold and Craig Tuerk in parallel selected specific RNA ligands for the T4 DNA polymerase [11]. The selected aptamers from the beginning have shown affinities and specificities comparable, or even superior in some cases, to those of their corresponding antibodies [12]. For example, an aptamer that recognizes interleukin (IL)-6 with a dissociation constant (*K*_d_) of 0.2 nM has been recently described [13]. Aptamers are gaining importance as therapeutics due to their potential advantages over cell-based products, such as recombinant proteins or antibodies. They can be chemically synthesized, which enables quick productions and versatile chemical modifications. High stability, lack of immunogenicity, flexible structure, and small size increase their penetration strength [14]. One of the major concerns regarding oligonucleotide-based therapies is plasma stability and their half-life in serum. Aptamers can be easily modified due to their chemical nature and therefore optimize their yield. Further, custom-tailored properties of interest can be added. Specific substitutions of OH residues at 2′ position of ribose for O-methyl or F groups significantly enhance stability in plasma [14,15]. In order to increase the strength of the bond generated between the aptamer and its target, non-natural aromatic hydrophobic modifications based on deoxyUridine (dU) analogous bases, such as Benzyl-dU (Bn-dU) and Naphthyl-dU (Nap-dU), can be added [16]. This latter example is the case of SOMAmers (Slow-Off rate Modified Aptamers) that present protein-like modified side chains, which confer them the feature of exposing fewer hydrogen bonds, fewer charge-charge interactions, and fewer total polar contacts [16]. Furthermore, conjugation of aptamers to cholesterol has shown to enhance their half-life and biological activity in vivo in several pre-clinical studies [17,18,19]. Moreover, the addition of clinically compatible carriers such as polyethylene glycol (PEG) prevents aptamer exclusion by renal filtration, which increases the time the aptamer is circulating inside the organism [14,20]. For instance, a PEGylated anti-MUC1 aptamer-doxorubicin conjugate showed a 6-fold increase in aptamer retention in circulation when compared to unPEGylated aptamers [21]. It is also noteworthy that as opposed to what might occur with antibodies, oligonucleotides are unlikely to trigger a T-cell immune response displaying a reduced antigenicity when compared to cell-based products. In what concerns clinical translation, short oligonucleotides can be easily chemically synthesized on a large scale in good manufacturing practice (GMP) with reduced costs as compared to proteins that are cell-based derived therapeutic agents. Furthermore, due to their oligonucleotidic nature, aptamers can be easily neutralized with their inherent antidote to reverse any undesirable effect that may arise [22,23]. Aptamers are chemically malleable molecules that can be conjugated with different types of therapeutic cargos in relatively straightforward chemical reactions [14,24].

Aptamers are selected by a biochemical method called SELEX which stands for Systematic Evolution of Ligands by EXponential enrichment. This selection procedure begins with a random library pool of many different oligonucleotide species (10^12–15^). Each species of the library contains a random region of variable length (5–50 nucleotides (nt)) flanked by two constant regions at the 3′ and 5′ end. The library will undergo several evolutionary rounds of selection before the binding species are enriched and dominate the library. Each round of selection consists of three main steps: *Binding*, where the library is incubated with the target in order to get rid of the unbound species; *Partition*, in which binder species are separated from the target; and *Amplification*, where binder sequences are amplified by PCR (polymerase chain reaction) and further transcribed in vitro if the desired result is an RNA aptamer. The amplified species will serve as a substrate library for the next round of selection. The entire SELEX process used to require from 9 to 15 rounds of selection, which implied several months of work, but having at the researchers’ disposal new tools, such as high-throughput sequencing (HTS), drastically reduces the number of rounds and therefore the amount of work to be done [24]. HTS facilitates the identification of already amplified species at early selection rounds counteracting the intrinsic bias of the procedure [24,25]. Over the last few years, HTS has become an indispensable tool for aptamer selection (HT-SELEX) [25,26,27,28,29]. Since the first aptamer described in 1990, the SELEX procedure has evolved and introduced variants to optimize the selection depending on the purpose and several SELEX methods have been described [14,24]. The selection method described by Gold’s and Ellington’s groups can be termed conventional SELEX [10,11]. CE-SELEX (capillary electrophoresis-SELEX) uses capillary electrophoresis to purify the binder species [30]. Microfluidic-SELEX (M-SELEX) combines the conventional selection method with a microfluidic system [31]. The introduction of microfluidic techniques has enhanced the efficiency of other SELEX methods such as CE-SELEX [32]. The Cell-SELEX procedure is performed with cells, and counter selection might be carried out with cells lacking the expression of the target on the cell surface [33]. In vivo SELEX carries out the rounds of selection in live animals [34]. Additionally, Toggle-SELEX is used to obtain cross-reactive aptamers between different animal species [35,36]. l-conformed enantiomer aptamers have been described as Spiegelmers [37]. The first Spiegelmer aptamers were described as d-adenosine and l-arginine l-conformed binding aptamers by Fürste’s group [38,39]. Tailored-SELEX is based on identifying short aptamers that exclude the terminal primer-binding sites as part of the recognition domain using bridge adaptors that overlap the ligates at 3′ and 5′ ends [40]. Synthetic L-mirror-imaged aptamers provide the advantage of not interacting with original nucleic acids and not being metabolized by the target cell as well as presenting higher nuclease-mediated degradation resistance [41]. Moreover, Spiegelmers have shown very weak immunogenic potential [42].

Through SELEX, aptamers can be isolated against molecules of almost every nature, e.g., lipids, vitamins, sugars, proteins, or even small molecules [14]. Indeed, hundreds of aptamers have been published to date for various different purposes [14,24]. Some of the described aptamers are currently undergoing clinical trials for the treatment of homeostasis, metabolic diseases, cardiovascular diseases, and cancer [43]. One of the most advanced aptamers in cancer treatment is the anti-nucleolin aptamer AS1411. This 26mer G-rich DNA aptamer has been described to bind the nucleolar protein nucleolin overexpressed on the surface of tumor cells. It exerts antitumor activity by inducing cytotoxic effects that induce apoptosis and it is now undergoing phase I and II clinical trials for the treatment of renal cell carcinoma and other solid tumors as well as for acute myeloid leukemia in combination with Cytarabine (https://Clinicaltrials.gov. NCT00881244, NCT00740441, NCT00512083, NCT01034410). Eyetech Inc^®^ and Pfizer^®^ have developed the first-in-class aptamer targeting the vascular endothelial growth factor (VEGF). This aptamer has been approved by the FDA (Food and Drug Administration) as Macugen in 2004, also known as Pegaptanib. It was the first molecule of its kind achieving FDA approval and nowadays is used for the treatment of AMD (age-related macular degeneration) [44]. Macugen is a 27 nt-long pegylated RNA aptamer which targets VEGF_165_ isoform in order to prevent choroidal neovascularization [44]. It is a 2′-fluoro pyrimidine and 2′-O-methyl purine modified aptamer with a deoxythymidine terminal cap to increase its stability [44]. Macugen is intravitreally administered at 0.3 mg/eye once every 6 weeks to treat AMD. Nowadays, Pegaptanib is being assessed in clinical trials for the treatment of different diseases, such ischaemic diabetic macular edema (MIDME) in phase IV or uveitis and choroidal and iris neovascularization in pilot or Phase I studies, respectively [43]. The majority of therapeutic aptamers described to date has been developed to treat cancer, and despite the fact that plenty of them are still providing preclinical data [24], some of these selected aptamers are currently undergoing clinical trials. NOX-A12, a Spiegelmer developed by Noxxon Pharma^®^ (Berlin, Germany) is a 45 nt-long aptamer that blocks the stroma cell-derived factor (SDF-1) or also known as CXCL12, a chemokine which promotes stem cell migration to the bone marrow enabling vasculogenesis, tumor growth, and metastasis [45,46]. This L-RNA aptamer is linked to a 40 kDa PEG unit at the 3′ end with no further modifications and is currently undergoing Phase II clinical trials for the treatment of chronic lymphocytic leukemia and multiple myeloma [43,46]. The most used aptamer for cancer treatment is known as AS1411, developed by Antisoma PLC^®^ and previously termed as AGRO100. This DNA aptamer recognizes the external domain of the membrane nucleolin affecting different cellular pathways such as NF-κ-B and Bcl-2 [47,48]. AS1411 was the first drug targeting nucleolin and the first aptamer in clinical trials for the treatment of different types of cancer. It is now undergoing phase II clinical trials for the treatment of metastatic renal cell carcinoma (RCC), non-small cell lung cancer (NSCLC), and acute myeloid leukemia (AML). The use of AS1411 has shown very encouraging results and has shown to be safe and well tolerated by patients [43]. One of the most important issues of new-generation drugs is their toxicity, and results from clinical trials suggest that the most advanced aptamers, Macugen and AS1411, exert minimal toxicity in patients [49,50]. New information regarding the adverse effects induced after long-term dosage of Pegaptanib is currently being gathered from a Phase IV study for the treatment of subfoveal choroidal neovascularization (NCT00845273).

## 3. Interfering RNAs

**Figure 1:**
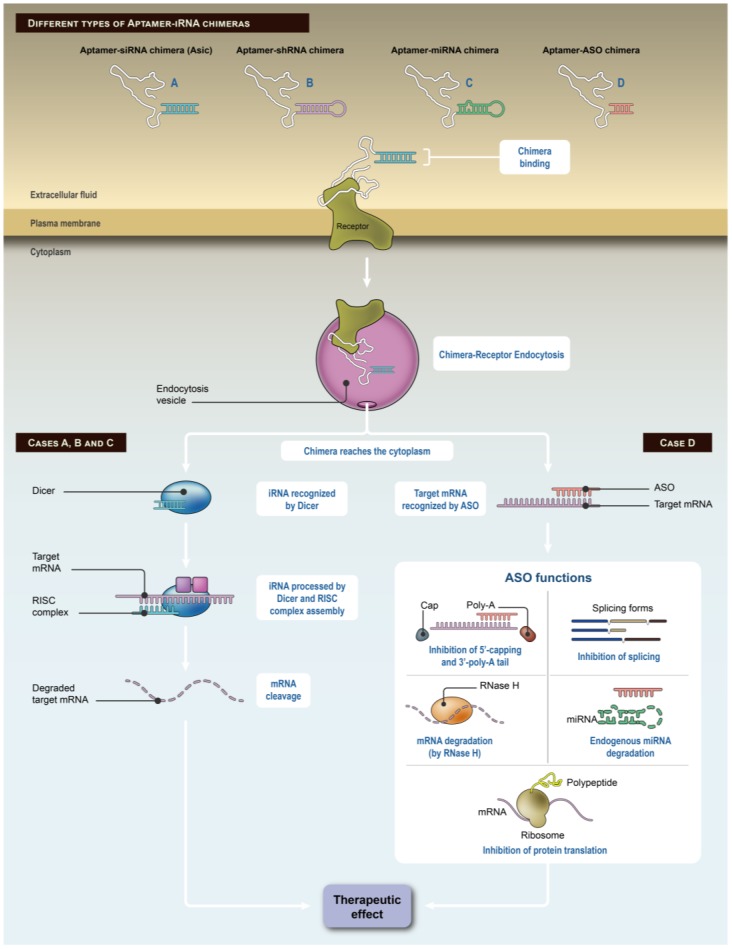
Aptamer-iRNA processing depending on the type of aptamer-iRNA chimera: (**A**) Aptamer-siRNA (AsiC); (**B**) Aptamer-shRNA; (**C**) Aptamer-microRNA, and (**D**) Aptamer-antisense oligonucleotide (ASO). Aptamer-iRNA chimera binds the aptamer receptor and upon engagement the chimera-receptor is embedded into an endocytosis vesicle. The chimera reaches the cytoplasm and in cases A, B, and C, the duplex iRNA is recognized by Dicer and loaded into Dicer and RNA-induced silencing complex (RISC) assembled to the target mRNA to be further degraded, and therefore exerts its therapeutic function. In case D, ASOs may exert their function by five different mechanisms: inducing RNAse H-mediated mRNA degradation, preventing 5’-capping and 3’-polyadenilation of the pre-mRNA, direct inhibition of protein translation, inhibition of splicing, or induction of endogenous micro-RNA degradation.

Interfering RNAs (iRNAs) are short RNAs that exert their biological activity by hybridizing with endogenous mRNA (messenger RNA) leading to the translational repression or degradation of the mRNA target gene. Briefly, as shown in Figure 1A, Dicer processes long RNA transcripts into short double-stranded RNAs which are loaded into the RNA-induced silencing complex (RISC) along with other proteins, such as Argonaute and transactivation response RNA-binding protein. This duplex iRNA consists of two strands, the passenger (sense strand, S), which is discarded, and the guide strand (antisense strand, AS), which will complementarily pair with the target mRNA sequence helped by the RISC. iRNA therapeutics comprise of the delivery of small interfering RNAs (siRNAs), short hairpin RNAs (shRNAs), microRNA mimics (miRNAs), and antisense oligonucleotides (ASOs). iRNA-based therapies provide a feasible alternative option when other anticancer therapies fail. Tens of iRNA-based therapies are currently undergoing clinical trials for different diseases, several of them in Phase III. Some of these iRNA-based therapies in clinical trials are aimed at treating multiple types of cancer [51]. Nevertheless, the application of such anticancer iRNA-based therapies requires major consideration regarding delivery, efficacy, and mostly, toxicity. Off-target effects of iRNAs owing to annealing of the guide strand to a non-target mRNA or undesirable immunogenic responses may trigger, in some cases, toxicity [51]. iRNA-based therapies encounter the major challenge of delivery as a result of evolution of defenses to maintain exogenous RNAs outside cells. Due to the intrinsic nature of iRNAs, lipid bilayers such as the plasma membrane provide very low or absent permeability to such molecules, which usually results in poor efficacy of iRNA activity [52]. Even though local administration of several iRNAs has been achieved, targeted inhibition of a given gene by systemic administration remains a difficult task to accomplish. Time and resources have been invested to solve such problems; early approaches began by conjugating iRNAs to cholesterol and human serum albumin (HSA) [53]. Lipid nanoparticles (LNPs) provided a feasible vehiculization for iRNA delivery as demonstrated by Zimmermann et al. in 2006 due to the masking effect exerted by LNPs that protects the iRNA from degradation [54]. Furthermore, iRNA encapsulation prevents undesirable responses owing to engagement of cellular toll-like receptors such as TLR-3, -7 or -8, or intracellular sensors such as retinoic acid inducible gene (RIG-I), among others [52]. Synthetic nanoparticles with polymeric materials, such as polyethyleneimine (PEI), have proved to efficiently deliver nucleic acids but their use often results in associated off-target effects [55]. Other synthetic nanoformulations have achieved efficient iRNA delivery, both in vitro and in vivo, reducing target mRNA expression up to 85% in human cells at a relatively low concentration (30 nM) [56]. Specific delivery of iRNAs to hepatocytes has been also accomplished by conjugation to *N*-acetylgalactosamine in order to target an asialoglycoprotein receptor (ASGPR) present in the liver. Weekly subcutaneous (SC) administration of siRNA-GalNac conjugate resulted in constant target silencing for nine months with no detected adverse effects [57]. Thus, due to the great necessity for interfering RNAs (iRNAs) to be targeted to specific cells to treat a given disease, the generation of aptamer-iRNA chimeras seems to be a very promising tool for cancer treatment. Here we focus the review on aptamer-based targeting of interfering RNAs, such as siRNAs, shRNAs, and ASOs as delivered cargos. Aptamer-iRNA chimeras are summarized in Table 1.

### 3.1. Therapeutic Approaches Using Aptamer-iRNA

#### 3.1.1. Aptamer-siRNA

The use of siRNA-based therapies is frequently limited by tissue-specific targeting and the conjugation with targeting agents increases their efficacy, lowering the doses which decreases the possibility of side effects, while widening the therapeutic window [24]. Aptamer-siRNA chimeras, that have been also recently coined AsiCs, are becoming a very useful tool for targeting gene-knockdown in cancer therapy [14,24]. The first approach to achieve this end was published by Levy and Ellington in 2006. They generated a construct consisting of a streptavidin-biotin-based conjugate to deliver anti-lamin A/C and anti-GAPDH siRNAs to prostate cancer cells. The conjugate comprises a streptavidin core bound to four biotin molecules covalently coupled to either the siRNA or the anti-PSMA aptamer A10. PSMA aptamer A10 presents flour (F) groups substituting the OH groups at the 2′ position of the ribose to increase its stability in plasma. The majority of the therapeutic RNA aptamers described to date and reviewed here show the same modification. The final construct was assembled by incubating streptavidin with biotin at 1:1 ratio of siRNA and PSMA that should render an average of two siRNAs and two PSMA aptamers per conjugate. They performed in vitro experiments adding the aptamer-siRNA chimera to the media at a final concentration of 45 nM (22.5 nM of the siRNA and 22.5 nM of the PSMA aptamer) or transfecting PSMA-positive LNCaP tumor cells at 45 nM of the siRNA as well. The results revealed that AsiC-mediated inhibition of target genes rendered gene expression at similar levels to that obtained by transfection, which demonstrated the advantage of aptamers as delivery agents [80]. That same year, a new class of such reagents was described by McNamara II generating the PSMA aptamer A10 with siRNAs as a single RNA molecule annealed to the siRNA guide strand. In this work, they used the PSMA-binding aptamer A10 to generate different aptamer-siRNA chimeras to target Plk1 and Bcl2 silencing in prostate PSMA-expressing cancer cells [58]. The use of PSMA-Plk1 (A10-Plk1) and PSMA-Bcl2 (A10-Bcl2) AsiCs resulted in specific binding, delivery, and silencing of target genes in PSMA-expressing cells in vitro. The experiments performed on PSMA-lacking prostate cancer cells, such as PC-3, did not show any silencing due to the absence of PSMA on the cell surface. In order to further demonstrate that the delivered gene silencing was being exerted in an aptamer-mediated manner, they mutated the A10 aptamer (A10mut-Plk1) and no binding nor silencing was detected in such controls. Moreover, intratumoral administration of A10-Plk1 AsiC in LNCaP-bearing athymic mice resulted in significant tumor regression when compared to the corresponding controls, such as PSMA-negative cells (PC-3) and A10mut-Plk1 [58]. Nevertheless, systemic administration of these new kind of reagents was needed to obtain more translational relevance and show their clinical feasibility. Three years later, the same group published an optimized version of the A10-Plk1 AsiC capable of targeting human PSMA-positive tumors when treated systemically [59]. They truncated the PSMA-binding A10 aptamer from 71 to 39 nt (A10-3.2) without impacting its affinity in order to ease its chemical synthesis. This new A10-3.2-Plk1 aptamer-siRNA chimera was further modified to increase its specificity and silencing activity. To that end, several modifications were introduced: the last nucleotide of the 3’ end of the siRNA duplex, which corresponds to a C, was mutated for a U in order to favor the loading of the guide (AS, antisense strand) silencing strand into RISC (wobble conformation); two uracils (U) were added to the 3′ end of the siRNA duplex; both strands of the siRNA duplex were swapped to provide 5′ terminal modifications of the silencing strand (swap conformation) to facilitate Dicer accessibility; and finally, the aptamer-siRNA chimera was integrated into a contiguous complete stem-loop sequence to simulate endogenous miRNA precursors (stem-loop conformation). While binding capacity of the optimized AsiC was comparable to the original one, the effect on gene silencing of both swap and stem-loop conformation was enhanced up to 99% silencing at 4 nM. Systemic administration of the optimized AsiC in its swap conformation in PSMA-expressing 22Rv1 (1.7) tumor-bearing athymic mice resulted in a significant reduction of tumor growth and Plk1 silencing. Furthermore, PEGylation of the optimized swap AsiC significantly increased in vivo half-life and inhibited tumor growth at substantially lower doses [59]. Using the same A10 PSMA aptamer, Pastor et al. in 2010 described an RNA aptamer coupled to a siRNA to target Nonsense-Mediated RNA decay (NMD) to tumor cells in order to enhance tumor antigenicity. NMD is a constitutive system in charge of controlling and degrading out-of-frame aberrant transcripts containing premature stop codons (PTCs). In the subcutaneous colon carcinoma CT26 murine model, the targeted inhibition of genes smg1 or upf2 significantly inhibited tumor growth when the targeted delivery of control siRNAs exerted minimal antitumor effect. Moreover, the introduction in this strategy of boosting agents such as 4-1BB co-stimulation further added antitumor effects. Within a context of metastatic melanoma, a PSMA transduced B16/F10 melanoma cell was used to assess whether this strategy was able to reach disseminated lesions, and the group treated with PSMA-smg1 siRNA exerted a more profound lung metastasis inhibition [60]. Soldevilla et al. recently developed a new chimera to treat CD40-expressing B-cell lymphomas by targeting NMD inhibition as well. The chimera consists of a CD40 RNA agonist aptamer coupled to a shRNA against one of the main NMD factors SMG-1. This immunotherapeutic chimera resulted in efficient NMD-targeted inhibition in CD40-expressing B-cell lymphomas while providing optimal conditions for cancer immunotherapy; activation of antigen-presenting cells, enhancement of tumor antigenicity, and bone-marrow aplasia recovery [68]. In 2012, Gilboa’s group published a useful set of rules to rapidly screen siRNA sequences for a given mRNA target suitable for conjugating with aptamers, maintaining its biological activity [81]. The inhibitory activity of siRNAs tends to decrease when conjugated to aptamers, probably due to the thermosensitivity of the non-cleavage-based displacement of the passenger strand of the siRNA duplex. RISC needs two steps: physical association of the proteins with siRNA duplex and the mRNA, and activation. Activation implies the displacement of the passenger strand, usually limiting the process. Displacement of the passenger strand can be achieved by cleavage or by a non-cleaving manner. The cleavage carried out by Argonaute 2 (Ago2) would not depend on the melting temperature (*T*_m_) of the siRNA duplex process, but when the passenger strand is removed in a non-cleaved manner by Ago 1, 3, or 4, it might be thermosensitive [82]. Gilboa’s group showed that displacement of the passenger strand would depend on the melting temperature of the siRNA duplex where siRNAs with low *T*_m_ would exert a higher biological activity. In this work, Berezhnoy et al. demonstrated that incorporation of 2′-fluoromodified pyrimidines affects the silencing activity of the siRNA as well as the *T*_m_ of the siRNA duplex. Aptamer-siRNA conjugation importantly affects the biological activity of the siRNA where the Aptamer-AS/S (sense, passenger strand) configuration results more detrimental than Aptamer-S/AS configuration. As mentioned above, the wobble conformation of the AsiC achieved by introducing a mutation on the 5′ end of the S strand (C→U) improved the inhibitory activity of the chimera without reaching the full effect of the native unwobbled siRNA. More importantly, they showed a correlation between the thermal stability and the conjugated siRNA inhibitory activity. They also demonstrated that the majority of the siRNAs tested with a calculated *T*_m_ lower than 50 °C maintained elevated levels of biological activity comparable to those of the native unconjugated siRNAs when siRNAs with a higher *T*_m_ exhibit lower biological activity in their conjugated form [81]. The same group generated a 4-1BB-mTORC1 AsiC to target mTORC1 inhibition in CD8^+^ tumor-specific *T* lymphocytes. This strategy enhanced their differentiation into CD8^+^ memory cells leading to a potent antitumor effect in B16 melanoma and 4T1 breast cancer tumor models [61]. Lieberman’s group described an AsiC consisting of EpCAM aptamer conjugated to PLK1 siRNA. This 2′-fluoro pyrimidine-containing EpCAM aptamer was linked to the siRNA at the 3′ end with different linkers also adding 3′-dTdT overhangs in order enhance its in vivo stability, reduce off-target effects, and limit innate immune system activation. This strategy, aimed at targeting the delivery of PLK1 siRNA to breast cancer, resulted in specific PLK1 knockdown in vitro and in human ex vivo cultured cells. They also achieved inhibition of mammosphere formation, which implies inhibition of proliferating tumor-initiating cells. In a triple-negative breast cancer MB-468 murine model, the subcutaneous administration of EpCAM-PLK1 siRNA chimera suppressed tumor growth [62]. Recently, Gilboa’s group has described a 4-1BB aptamer IL-2R alpha (CD25) siRNA chimera for the treatment of breast cancer. This AsiC was systemically administered in flank-sited 4T1-bearing balb/c mice resulting in a potent antitumor response that potentiated the effect of irradiated B7-1 and MHC-II-expressing 4T1 cells as vaccines. This strategy significantly reduced tumor growth as well as downregulation of IL-2 signaling and the promotion of a memory-like phenotype [63]. This same group, and following the same rationale, generated a 4-1BB smad4 AsiC to target TGFβ inhibition signaling in CD8^+^ cells. In this case, the systemic administration of such AsiC in combination of either radiation (12Gy) or irradiated B7-1 and MHC-II 4T1 cell-based vaccine enhanced their antitumor immunity [64]. It is known that STAT3 works as a regulator favoring tumor formation in different tumor types including leukemia, colon and renal carcinomas, breast cancers, and glioblastoma multiforme (GBM) [83,84]. Herrmann et al. described an AsiC consisting of the previously described aptamer against the cytotoxic T-lymphocyte antigen 4 (CTLA-4) [85] and a STAT3 siRNA. They show that both local and systemic administration of the CTLA-4-STAT3 AsiC drastically reduced tumor associated regulatory *T* lymphocytes (Tregs) in various tumor models, as well as a potent inhibition of tumor growth and metastasis [65]. Franciscis’ group have recently published a targeted STAT3 inhibition strategy using the Gint4.T Platelet-derived growth factor receptor β (PDGFRβ) antagonizing aptamer previously described by the same group [86]. They use the Gint4.T aptamer as a targeting agent conjugated to a STAT3 siRNA to target STAT3 inhibition to glioblastoma cells [66]. In the work published by Esposito et al., the Gint4.T-siRNA STAT3 chimera was able to decrease cell viability and migration in vitro in brain tumor cell lines and more importantly inhibiting tumor growth, decreasing the tumor burden, and inhibiting angiogenesis in immunodeficient athymic U87MG tumor-bearing mice [66]. A new approach has been recently described consisting of a triple AsiC for the treatment of breast cancer. This new chimera was formulated as an EGFR (endothelial growth factor receptor) siRNA disposed between two aptamers, HER2 and HER3 [67]. This AsiC termed H2EH3 arrested cell cycle and induced apoptosis in vitro as determined by upregulating cleaved caspase-3 and also p21 in BT474 and SKBR3 HER2^+^HER3^+^ cells. At the same time, this triple targeting strategy knocked down the three genes (EGFR, HER2, and HER3) at a protein level. Using BT474-bearing athymic nude mice as xenograft murine model groups treated intratumorally with the H2EH3 AsiC reduced tumor size 1.5-fold when compared to the administration of HER2 and HER3 aptamer mixed with EGFR siRNA, and 7-fold when compared with the rest of controls. Following the same type of experiment, but in this case with the triple AsiC, it was intravenously administered and the comparison with the mixture control rose to a 4-fold change, while compared to the rest of control maintained the same difference of 7-fold. It is important to note that imaging experiments revealed no detectable toxicity. ELISA experiments showed no IFN-gamma production of triple AsiC-treated human peripheral mononuclear cells (PBMCs) indicating that it is likely not to exert any undesirable innate immune response [67].

The size of the delivered RNA might provide additional troubleshooting at the time of targeting but, as recently demonstrated by Porciani et al., the RNA payload does not affect the aptamer-based targeted delivery of such molecules [87]. They used Waz and C10.36 aptamers to specifically in vitro deliver both short and large RNAs to canine CLL17 B-cell lymphoma cells and canine CLGL-90 leukemia *T* cells. They use a fluorescence “broccoli”-based tracking system to monitor the stability of intracellular RNAs. Broccoli is a variant of the Spinach fluorogenic RNA aptamer which when complexed with a specific small molecule fluorophore emits fluorescence. The structure of the aptamer-fluorophore complex has been used in this cited work as a sensor to assess the maintenance of the original folding structure and measure RNA degradation. They demonstrate that the aptamer nanostructure they were using remained highly stable at early stages of endocytosis. They also showed that aptamer-RNA constructs remained stable and functional inside the endosome for several hours, which might facilitate a long-lasting controlled release to the cytosol to further exert its function [87]. This work opens the door to the designing of new strategies that favor the translocation of a given cargo (iRNAs, peptides, drugs, etc.) from the endosome to the cytosol, thereby enhancing the efficacy of aptamer-mediated targeting approaches.

#### 3.1.2. Aptamer-shRNA

As it has been mentioned, another class of interfering RNAs is shRNA. shRNAs are short synthetic iRNA molecules with a hairpin loop-turn that silence target gene expression or target mRNA degradation. shRNAs as can be seen in Figure 1B are recognized and processed by Dicer inside the cytoplasm and in assistance with RISC, Ago, and other RNA-binding proteins, the guide strand is hybridized to the complementary mRNA molecule to its further cleavage and degradation resulting in gene knockdown. Delivery of shRNA into cells is usually achieved using different types of vectors, which in the majority of the cases implies undesirable off-target and immunogenic side effects for therapy [88]. Therefore, efficient delivery and targeting of shRNAs make aptamers solid candidates to accomplish this aim. Following the line of siRNAs, they can be conjugated to aptamers as delivery and targeting agents [24]. One pioneer approach in shRNA delivery using aptamers for cancer treatment was described by Kim et al. in 2010 studying the synergy between doxorubicin (DOX) and shRNA-mediated Bcl-xL gene knockdown. They generated aptamer-conjugated polyplexes (APs) consisting of DOX-intercalated polyethylene glycol (PEG)-engrafted PEI as nanoparticles containing cl-xL specific shRNAs. In vitro studies showed a potent Bcl-xL downregulation after 48 h which, in addition to DOX effect, exerted an excellent antitumor effect [69]. Further reading in aptamer target nanoparticles can be found in these recent reviews [14,89,90,91]. A work published by Ni et al. demonstrated that a PSMA-DNAPK (DNA-activated protein kinase) shRNA chimera could be used as a therapeutic agent for prostate cancer [70]. DNAPK is a key factor in DNA repair systems, which is strongly needed after ionizing radiation (IR)-induced DNA damage identified by siRNA library screening. In this work, Ni et al. showed that DNAPK PSMA-based targeted inhibition sensitized LNCaP prostate cancer cells to ionizing radiation, as shown in in vitro MTS assays where the aptamer-shRNA chimera increased cell death after IR cycles but not the controls. They assessed its efficacy in LNCaP and PC3 prostate tumor models and found that the combination of radiation with the aptamer-shRNA chimera significantly increased tumor response, more effectively in LNCaP than PC3 models. LNCaP tumors receiving the combinatorial therapy needed 10 weeks to reach the same tumor volume as those treated with the proper controls. Furthermore, ex vivo immunohistochemistry imaging showed that PSMA-expressing human tissue was susceptible to PSMA-DNAPK shRNA treatment reducing DNAPK expression by 25% when compared to control treatments [70]. In 2015, Askarian et al. published the targeted delivery of Bcl-XL shRNA in an aptamer nanoparticle-based manner. They conjugated the anti-nucleolin AS1411 DNA Aptamer to polyethylenimine and poly L-Lysine nanoparticles to specifically deliver a Bcl-XL shRNA-encoding plasmid to lung cancer cells. They achieved a significant decrease in Bcl-XL expression at both mRNA and protein levels. The in vitro experiment revealed induction of apoptosis in nucleolin-expressing A549 lung cancer cells but not in nucleolin-negative fibroblast cell line L929 [71].

A new strategy has been generated to effectively transfect shRNAs to cancer cells using the anti-nucleolin aptamer AS1411 as a targeting agent. The AS1411 aptamer was covalently conjugated to polyamidoamine (PAMAM) through an amide bond to increase transfection efficacy and coupled to a shRNA to inhibit Bcl-xL in target cells. The system was evaluated in A549 nucleolin-expressing lung cancer cells using L929 fibroblasts as a negative control. The results showed that the AS1411-PAMAM-shRNA conjugate efficiently targeted nucleolin-expressing cells. The chimera knocked-down the expression of Bcl-xL protein as demonstrated by western-blot (WB) and selectively induced apoptosis in nucleolin-expressing cells but not in control cells as shown by anexin V and PI (propidium iodide) experiments [72].

#### 3.1.3. Aptamer-microRNA

MicroRNAs (miRNAs) are 19 to 24 nt long non-coding RNAs that function as silencers or post-transcriptional regulators of gene expression. They are encoded by nuclear DNA and exert their activity by pairing with complementary sequences within their mRNA target [92]. Inside the nucleus, genes encoding miRNAs, usually located within intronic regions, are transcribed by RNA polymerase II into a stem-loop shaped primary miRNA (pri-miRNA). Drosha accompanied by its cofactor DGCR8 recognize pri-miRNA and cleave the 3′ and 5′ end to render a pre-miRNA which will be exported to the cytosol through nuclear pores. Once in the cytoplasm, RNase III Dicer and TRBP (TAR RNA binding protein) cleave the terminal loop resulting in a miRNA duplex. As shown in Figure 1C, the miRNA duplex will be recognized by RISC (RNA-induced silencing complex) and will be processed by the Argonaute (AGO) complex of proteins along with several cofactors to eliminate the disposable strand and generate a mature miRNA. This mature miRNA engages fully complementary sequences usually at the 3’-UTR of the target mRNA leading to its degradation, but when complementarity is not complete, silencing might be prevented by ribosomal blockade [8]. Some of the micro-RNAs are known as oncomiRs with pro-tumorigenic and tumor suppressor functions.

Tumor suppressor miRNAs have gained importance in cancer therapies and targeted delivery of different families of tumor suppressor miRNAs has aroused great interest in recent years. Esposito et al. described the generation of an aptamer-miRNA chimera to target the delivery of let-7g miRNA to tumor cells. They used the GL21.T aptamer which blocks the activity of the oncogenic receptor tyrosine kinase Axl and demonstrated the specificity of the GL21.T-let aptamer-miRNA chimera targeting by increased accumulation of let-7g in Axl-expressing cells A549 but not in MCF-7 Axl-lacking cells. After systemic administration of the aptamer-miRNA chimera in A549 tumor-bearing immunodeficient mice, there was down-regulation of let-7g target HMGA2 as well as inhibition of tumor growth [73]. The same group developed a strategy using the same aptamer conjugated this time to the miR-212 to deliver it into A549 NSCLC (non-small cell lung cancer) cells. Laboni et al. demonstrated that GL21.T-miR-212 increase caspase activation in NSCLC, and the enhanced effect of such a strategy when combined with TRAIL therapy as adjuvant [74]. Another work used the transferrin receptor aptamer (TRA) to deliver miR-126 to endothelial and breast cancer cells. They generated a chimera consisting of the TRA conjugated through a sticky bridge to the pre-miR-126. It effectively delivered the pre-miR-126 to human endothelial cells and to MCF7 breast cancer cells in vitro. It inhibited the effect of its target VCAM-1 reducing proliferation of MCF7 cancer cells as well as decreasing paracrine endothelial cell recruitment in the same breast cancer in vitro model. It has to be noted that the effect achieved by this strategy is comparable to that obtained by liposome-mediated transfection of miR-126-3p mimic [75].

Novel anticancer therapies usually follow first-line treatments such as surgery, radiotherapy, and/or chemotherapy. In the majority of cases, radiotherapy and chemotherapy result in hematopoietic toxicity exerting potent myeloid cell suppression. Other approaches have been described to restore radiotherapy-induced myelosuppression as mentioned earlier in this text [68]. A new aptamer-based strategy has been recently published to achieve this goal, attenuating the hematopoietic toxicity induced by chemotherapeutic drugs, such as carboplatin and 5′ fluorouracil (5-FU) in hematopoietic stem/progenitor cells (HSPCs). They generated a chimera consisting of the c-Kit aptamer conjugated to mir-26a mimic to restore Bak1 (Bcl-2 antagonist/killer 1) in HSPCs. They demonstrated the importance of restoring BAK1 in chemotherapy-induced myeloablation and also after bone marrow transplantation with the c-Kit-miR26a mimic chimera. Moreover, in vivo experiments in NOD scid gamma NSG mice showed that systemic administration of the aptamer-microRNA chimera protects individuals from carboplatin and 5-FU while inhibiting tumor growth in a MDA-MB-231 human breast cancer tumor model [76].

#### 3.1.4. Aptamer-Antisense Oligos (ASOs)

Antisense oligonucleotides are synthetic single-stranded oligonucleotides that are 18 to 21 nt long and complementary to the mRNA target gene. ASOs are aimed at modulating the expression of a given gene at the mRNA level by interfering with its function. ASOs exert their inhibitory activity by four different mechanisms [93] as shown in Figure 1D, being the RNAse H activation, the most important knockdown system. RNAse H is an endonuclease that recognizes and degrades RNA heteroduplexes thus cleaving the target mRNA leading to its downregulation [94]. This ASO-mediated inhibitory effect may provide inhibitions of up to 95%. Given the ASO-mRNA specific recognition and therefore impeding the interaction with other nucleic acids, protein complexes, and other key factors for mRNA processing, splicing regulation is another important ASO-mediated mechanism of action. mRNA splicing requires the excision of introns by fine-tuned action of spliceosomes and the use of ASOs to recognize and bind to specific sequences needed for splicing, prevents binding of the required factors and therefore intron cleavage downregulating the expression of the mature form of the target protein [95]. ASO-mediated mRNA splicing regulation is not always aimed to inhibit such mechanisms but to correct them from aberrant forms, namely what occurs with β-thalassemia. In this specific disease, ASO amends the defective splicing that causes β-thalassemia restoring the functional protein [96]. Translation inhibition is a further ASO mechanism of action recognizing and binding to the initiating codon of the target mRNA physically impeding protein translation [93]. Finally, the last ASO-mediated mechanism of action is preventing the 5′-end capping or 3′-end polyadenylation of the pre-mRNA that leads to destabilization and mRNA degradation [95]. There exists a subtype of ASO-mediated mode of action that consists of the degradation of endogenous micro-RNAs usually performed by antagomiRs (anti-miRs). As will be described below, anti-miRs act by pairing with miRs to be further degraded by RNAses and inhibit the micro-RNA function. As is the case with other iRNAs, delivery is a major issue encountered when designing clinically feasible ASO-based therapies, especially when ASOs need to reach the cell nucleus to exert their function, which has become an important challenge in iRNA delivery. Several modifications have been introduced to improve their availability and cellular uptake, but considerable undesired side effects tend to be present in ASO-based therapies [93].

Concerning aptamer-ASO chimeras in the context of cancer therapy, Sullenger’s group described a few years ago an aptamer-splice-switching antisense chimera able to modify the splicing inside the cell nucleus. They generated a chimera consisting of the anti-nucleolin AS1411 aptamer conjugated to a splice-switching oligonucleotide (SSO, a subtype of ASO) towards luciferase. They tested this construct in PC3 prostate cancer cells containing a luciferase reporter system (PC3/Luc 705 cells) with a premature stop codon within the luciferase gene to prevent its translation. When PC3/Luc 705 cells were treated with the AS1411-Luc SSO, the repaired luciferase mRNA percentage increased up to 13%, demonstrating that aptamer-SSO chimeras can indeed alter pre-mRNA splicing [78]. This important work demonstrates once again that aptamers are a very suitable tool for iRNA targeting and delivery not just to the cytoplasm but to the cell nucleus using a specific fine-tuned aptamer-iRNA system.

Antagomirs, also called anti-miRs, are synthetic oligonucleotides designed to directly block the mature form of miRNA, impeding their engagement with target mRNA. The aim of antagomirs is to block the effect of the miRNA thus inhibiting the transcriptional repression and degradation of the mRNA induced by a given miRNA [8]. AntagomiRs or anti-miRs are a subtype of ASOs and therefore they may undergo the same chemical modifications in order to enhance their activity. Some of these modifications are the use of phosphorothioate backbone that increases their half-life, locked nucleic acids (Lna) or 2′ modifications on the ribose sugar such 2′-O-methyl and 2′-O-metoxyethyl to enhance target binding and stability, while reducing undesirable toxic effects [97,98].

It has been demonstrated that miRNA blockade by complementary base-pairing using anti-miRs restores oncomiR-mediated proliferative activity in cancer cells [99]. There exists a necessity of finding non-toxic carriers to target anti-miRs only to cancer cells and therefore decrease off-target effects associated with systemic administration of such iRNAs. Catuogno et al. generated two different constructs to deliver the tumor suppressor anti-miR 222, one conjugated to the Axl aptamer (GL21.T), and another coupled to the antagonistic aptamer against the platelet-derived growth factor receptor β (PDGFRβ). Both aptamers functioned as effective tumor delivery agents in, respectively, Axl-expressing and PDGFRβ-expressing U87MG cells. They generated a GL21.T-222 aptamer chimera based on the sticky junction sequence proposed by Rossi’s group in another recent publication [100]. Furthermore, they combined the action of both anti-miR 10b and anti-miR 222 within the same GL21.T bi-modular sticky aptamer chimera and it reduced the intracellular levels of the two corresponding proteins. U87MG xenograft was systemically treated with the GL21.T-222 chimera resulting in significant reduction of tumor burden as well as higher concentration of the conjugate in the excised tumor masses [79].

Combining both therapies (aptamer-based targeting of miRNAs and antagomirs) would potentiate antitumor effects regulating the expression of mRNAs, enhancing their activity and restoring the function blocked by miRNAs in the same target cell. For example, Esposito et al. further combined both strategies using the Axl aptamer and the PDGFRβ for the delivery of microRNA 137 and antagomir 10b respectively. They generated two chimeras, GL21.T-miR-137 and Gint4.T-anti-miR-10b, to target the delivery of both iRNA to glioma stem cells. This combined targeted delivery was able to disrupt tumor sphere formation in vitro and inhibit cancer stem cell proliferation crossing the blood-brain barrier in the context of an in vitro model as well [77].

Aptamers have shown great potential as therapeutic agents by either blocking and/or activating receptors depending on the purpose, as well as carriers and targeting agents [14,24]. Due to their properties and their malleable structure, novel aptamer-based therapeutic approaches have been developed over the last few years. It should be noted that aptamers may work as antagomirs and can be used as microRNAs-blocking agents themselves, as demonstrated by Sczepanski et al. in a published work where they use enantiomer RNA aptamers to inhibit the oncogenic effect of miR-155. They isolated an L-RNA aptamer that recognizes the pre-miR-155 and blocks the activity of miR-155. These new L-conformer RNA aptamers that recognize the stem-loop of pre-microRNAs are termed as “aptamiRs” and bind their target through specific three-dimensional conformation interactions rather than classic Watson–Crick base pairing [101].

**Figure 2:**
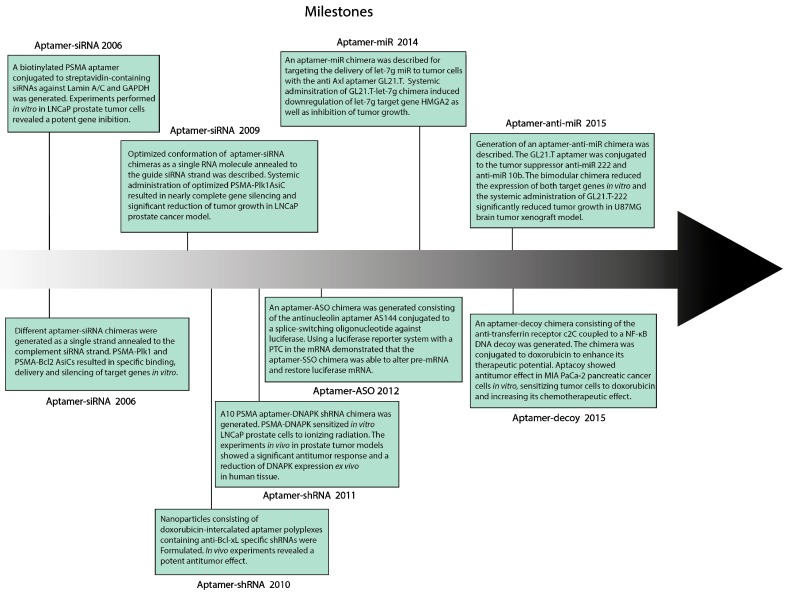
Milestones of each type of aptamer-iRNA chimeras for cancer treatment. PTC = premature stop codon; SSO = splice-switching oligonucleotide.

It is worth mentioning that there are other types of therapeutic RNAs beyond iRNAs that can be conjugated to aptamers for their delivery to treat cancer as well as other diseases. Aptamers can be coupled to other aptamers, for instance, to target T-cell costimulation or NK activity to the tumor site [102,103,104,105,106]. They can be conjugated to DNA or peptide decoys in order to inhibit a transcription factor function [107,108] or to small-activating RNAs (saRNAs) [109,110] to enhance the expression of a given gene. This issue gathers the most relevant information on aptamer-iRNA chimeras and decoys, despite being presented in their majority as double-stranded DNA molecules, are ODNs that specifically bind to a given transcription factor inhibiting its inherent function. Aptamer-Decoy chimeras needed to be mentioned as well in this review. Porciani et al. presented a work not long ago where they couple the anti-human transferrin receptor aptamer (c2C) to a NF-κB DNA decoy. NF-κB is a survival factor usually linked to acquired chemoresistance [111]. They generated what can be called “aptacoy” and its therapeutic potential was enhanced by conjugating the chimera to the chemotherapeutic drug Doxorubicin [107]. It was demonstrated that tumor-targeted co-delivery of NF-κB decoy and Doxorubicin was mediated by the transferrin receptor aptamer. The in vitro antitumor effect in MIA PaCa-2 pancreatic cancer cells was owed to decoy-based NF-κB inhibition, which leads to sensitization to Doxorubicin, ultimately increasing its chemotherapeutic effect in the target cells [107]. STAT3 is a transcription factor with important immune-regulatory activity in tumor and tumor microenvironment-immune cells facilitating angiogenesis, tumor cell proliferation, and survival [83,112]. There is no accessibility to FDA approved STAT3 inhibitory drugs, but different approaches based on small molecules and oligonucleotides have been described in the last years [113,114,115,116,117]. Kortylewski’s group has pioneered ODN decoy-based STAT3 inhibition in both leukemias and B-cell lymphomas. In this latter work, they demonstrated that decoy-based targeted inhibition of STAT3 induced STAT3 inhibition, tumor cell death, and immune system activation through TLR9 signaling [118]. As commonly known, FOXP3 is a key transcription factor for the maturation of regulatory T lymphocytes (Tregs). The presence of such cells within the tumor usually favors tumor growth which implies the necessity of FOXP3 inhibition to improve cancer therapies. The P60 compound is a peptide able to inhibit FOXP3 function, impeding the transcription of FOXP3 target genes that has been proven to enhance the efficacy of anti-tumor vaccines [119]. Nevertheless, a work was recently published where the targeted transient inhibition of FOXP3 was achieved by generating a new chimera consisting of the anti-CD28 RNA aptamer coupled to the P60 peptide. Treatment with the chimera resulted in similar outcome as the free peptide, reducing the effective dose up to 800 times, widening the therapeutic window as well as likely reducing putative off-target effects of the systemic administration of the free peptide [108]. A couple of works have been recently published where aptamers were conjugated to saRNAs to treat cancer [109,110]. saRNAs are double-stranded RNA molecules that recognize gene promoter regions by base-pairing, and thus upregulating the transcription of the target gene [120,121]. Li et al. described an aptamer-saRNA chimera consisting of the above mentioned PSMA-binding aptamer A10-3.2 conjugated to saRNAs that increase Dihydro-pyrimidinase-like 3 (DPYSL3) gene expression. DPYSL3 has been identified as a metastatic suppressor gene in prostate cancer [109]. Using this new therapeutic approach, they demonstrated that treating human PSMA-positive cancer cells in vitro with A10-3.2-saV2-9RNA chimera reduces their motility and increases DPYSL3 expression. Moreover, treatment of C4-2 and LNCaP prostate cancer cells using an orthotopic xenograft tumor model in athymic mice with the aptamer-saRNA chimera revealed a significant suppression of distal metastasis, as well as enhanced DPYSL3 expression in xenograft tissues [109]. CCAAT/enhancer-binding protein-α (C/EBPα) downregulation has been demonstrated to enhance aggressiveness of pancreatic ductal adenocarcinoma (PDAC) [122]. C/EBPα expression inhibits tumor formation in PDAC and aptamer-mediated targeted activation of such genes might provide a feasible approach in PDAC cancer therapy. Rossi’s group generated an aptamer-saRNA chimera to this end. They selected PDAC specific RNA aptamers through Cell-SELEX to use them as delivery agents and conjugated them to C/EBPα saRNAs [110]. This chimera enhanced C/EBPα expression in PDAC cells in vitro, and significantly abolished cell proliferation. They tested this approach in PDAC PANC-1 and AsPC-1 mouse xenografts and the systemic administration of the chimera significantly reduced tumor burden in mice [110]. A timeline is shown in Figure 2 describing different types of aptamer-iRNA chimera achievements in the context of cancer treatment.

## 4. Concluding Remarks

iRNA-based therapies arose as one of the most promising anticancer therapies, but they encountered several problems at the time of translation into the clinic. iRNAs have shown poor biodistribution, low tissue and cell penetrability, and lack of specificity for the target cell. Aptamers have gained remarkable importance as therapeutics over the last few years. Due to their small size, high affinity for their targets, and high tissue penetrability, aptamers are a very suitable tool for iRNA targeting and delivery. Aptamer-iRNA chimeras might open a new era for iRNA-based therapies, enhancing their specificity and biodistribution, reducing off-target effects, and therefore facilitating their translation into the clinic. Here we have gathered the most important information on aptamer-iRNA chimeras for cancer treatment described in the last years.

## Figures and Tables

**Table 1:** Summary of aptamer-based targeted delivery of interfering RNAs for cancer treatment. NSCLC = Non-small cell lung cancer.

Type of Chimera	Aptamer	iRNA/Target	Type of Malignancy	Model	Reference
**Aptamer-siRNA**	PSMA	PLK1 and BCL2	Prostate cancer	LNCaP	[58]
	PSMA	PLK1 and BCL2	Prostate cancer	22Rv.1 (1.7)	[59]
	PSMA	SMG-1 and UPF2	Colon carcinoma and metastatic melanoma	CT26 and B16/F10	[60]
	4-1BB	mTORC1	Melanoma and breast cancer	B16 and 4T1	[61]
	EpCAM	PLK1	Breast cancer	MDA-MB-468	[62]
	4-1BB	IL-2Rα	Metastatic breast carcinoma	4T1	[63]
	4-1BB	Smad 4	Metastatic breast carcinoma	4T1	[64]
	CTLA-4	STAT3	T-cell Lymphoma	Karpas299	[65]
	PDGFRβ	STAT3	Glioblastoma	U87MG	[66]
	HER2 and HER3	EGFR	Breast cancer	BT474	[67]
**Aptamer-shRNA**	CD40	SMG-1	B-cell lymphomas	A20	[68]
	PSMA	Bcl-xL	Prostate cancer	LNCaP	[69]
	PSMA	DNAPK	Prostate cancer	LNCaP	[70]
	AS1411	Bcl-xL	Lung cancer	A549	[71]
	AS1411	Bcl-xL	NSCLC	A549	[72]
**Aptamer-miRNA**	Axl	let-7g/HMG2	NSCLC	A549	[73]
	Axl	miR-212	NSCLC	A549	[74]
	TRA	pre-miR-126/VCAM-1	Breast cancer	MCF7	[75]
	c-Kit	miR-26a mimic/BAK1	Breast cancer	MDA-MB-231	[76]
	Axl	miR-137	Glioblastoma	U87MG	[77]
**Aptamer-ASO**	AS1411	Luciferase	Prostate cancer	PC3/Luc 705	[78]
**Aptamer-anti-miR**	Axl and PDGFRβ	anti-miR-222 and anti-miR 10b	Glioblastoma	U87MG	[79]
